# Exploring the epidemic transmission network of SARS in-out flow in mainland China

**DOI:** 10.1007/s11434-012-5501-8

**Published:** 2012-11-08

**Authors:** BiSong Hu, JianHua Gong, Jun Sun, JiePing Zhou

**Affiliations:** 15501 Geography and Environment Department, Jiangxi Normal University, Nanchang, 330022 China; 25501 State Key Laboratory of Remote Sensing Science, Institute of Remote Sensing Applications, Chinese Academy of Sciences, Beijing, 100101 China; 35501 Key Laboratory of Poyang Lake Wetland and Watershed Research, Ministry of Education, Jiangxi Normal University, Nanchang, 330022 China; 45501 Zhejiang-CAS Application Center for Geoinformatics, Jiaxing, 314100 China

**Keywords:** SARS, epidemic spread, in-out flow, transmission network, characteristic parameter

## Abstract

The changing spatiotemporal patterns of the individual susceptible-infected-symptomatic-treated-recovered epidemic process and the interactions of information/material flows between regions, along with the 2002–2003 Severe Acute Respiratory Syndrome (SARS) epidemiological investigation data in mainland China, including three typical locations of individuals (working unit/home address, onset location and reporting unit), are used to define the in-out flow of the SARS epidemic spread. Moreover, the input/output transmission networks of the SARS epidemic are built according to the definition of in-out flow. The spatiotemporal distribution of the SARS in-out flow, spatial distribution and temporal change of node characteristic parameters, and the structural characteristics of the SARS transmission networks are comprehensively and systematically explored. The results show that (1) Beijing and Guangdong had the highest risk of self-spread and output cases, and prevention/control measures directed toward self-spread cases in Beijing should have focused on the later period of the SARS epidemic; (2) the SARS transmission networks in mainland China had significant clustering characteristics, with two clustering areas of output cases centered in Beijing and Guangdong; (3) Guangdong was the original source of the SARS epidemic, and while the infected cases of most other provinces occurred mainly during the early period, there was no significant spread to the surrounding provinces; in contrast, although the input/output interactions between Beijing and the other provinces countrywide began during the mid-late epidemic period, SARS in Beijing showed a significant capacity for spatial spreading; (4) Guangdong had a significant range of spatial spreading throughout the entire epidemic period, while Beijing and its surrounding provinces formed a separate, significant range of high-risk spreading during the mid-late period; especially in late period, the influence range of Beijing’s neighboring provinces, such as Hebei, was even slightly larger than that of Beijing; and (5) the input network had a low-intensity spread capacity and middle-level influence range, while the output network had an extensive high-intensity spread capacity and influence range that covered almost the entire country, and this spread and influence indicated that significant clustering characteristics increased gradually. This analysis of the epidemic in-out flow and its corresponding transmission network helps reveal the potential spatiotemporal characteristics and evolvement mechanism of the SARS epidemic and provides more effective theoretical support for prevention and control measures.

The epidemic spread of infectious diseases is influenced by many factors, such as viral pathogenic characteristics, spatial-temporal range and distributions of epidemic outbreak, prevention and control measures, and population social activities; therefore, the epidemic spread mechanism is the focus of research in pathology, epidemiology, medical statistics, spatial information science, and sociology, among other fields. Research on epidemic spread could be classified into three types: (1) the macroscopic analysis of the statistical results of the epidemic spread; (2) the microscopic analysis of the interaction mechanism between individuals; and (3) the analysis of an epidemic spread in a macroscopic area caused by microscopic interactions between individuals.

A novel coronavirus-causing respiratory infectious disease, Severe Acute Respiratory Syndrome (SARS) induces fever, dry cough, chest tight, respiratory failure, and other symptoms as primary symptoms. SARS has spread pathways that include droplet transmission, contact with respiratory secretions and close spatial contact with infected cases, and SARS is recognized as a considerable threat to human health. Its outbreak and spread can cause significant human morbidity and mortality and directly influence social stability and economic development. The global spread of the SARS epidemic included 8422 cases with over 900 deaths widely distributed among 32 countries. The SARS epidemic in mainland China broke out in November 2002 and ended in July 2003, and SARS affected 26 provinces and municipalities, including 5327 cases with over 340 deaths. The SARS epidemic spread had significant regional differences in the distributions of the infected cases, and indicated obvious spatial-temporal characteristics and direct or indirect relationships among environmental, societal and economic factors, among others.

Recent research has focused on the macroscopic analysis of the spatial-temporal patterns of the SARS epidemic, such as the theoretical modeling of the SARS epidemic [1–3], analysis of the dynamic mechanism of the SARS epidemic process [4,5], statistical analysis of the epidemiological data [6], spatial-temporal statistical analysis of the distribution characteristics of the SARS epidemic, and autocorrelation and heterogeneity [7–11]. This retrospective and macroscopic research helps thoroughly recognize the spread mechanism of existing infectious diseases on a spatial-temporal scale and provides some probable scientific basis for researching other unknown infectious diseases. However, this research does not explain the spread characteristics from one individual to another or consider the interaction mechanism between individuals and regions. Some microscopic research, such as the simulation and prediction of the SARS epidemic process based on system dynamics and a multi-agent system [12,13] and simulation of the spread mechanisms based on models of small-world network and scale-free network [14,15], has concentrated on the activity rules of individuals and influences of control parameters of the epidemic spread mechanism; however, character transformation between individuals and influences of individual spatial activity during the epidemic process have rarely been considered.

In general, epidemiological data applied to research on epidemic spread mainly include confirmed infected cases and those in close contact with infected individuals. During the entire epidemic process, susceptible individuals first became infected, then became infectious, received treatment after the symptoms appeared and, finally, recovered. There are many epidemic characters for individuals. Meanwhile, there are corresponding spatial locations for the various individual characters and the spatial-temporal changes of these individuals during the process of susceptible-infected-symptomatic-treated-recovered cause different infectious strengths and ranges. From another perspective, regions have inputs and outputs of the epidemic spread according to the location changes of individuals with various epidemic characters, and there are interaction mechanisms between individuals and regions. Similar to other complex networks that exist in the real world, such as gene networks in biological systems, computer networks and social networks, the interactions between individuals and regions form a complex dynamic network of epidemic spread, and by analyzing the structure and characteristics of the epidemic spread network, some potential spread mechanisms may be recognized and shown to be the driving effects of various factors in the SARS epidemic.

Considering the location changes of individuals according to character transformation based on the SARS epidemiological investigation data in mainland China from 2002 to 2003, the in-out flow of the SARS epidemic was defined in this paper according to the three typical location data of the individuals (working unit or address, onset location and reporting unit), and a transmission network of the SARS epidemic caused by the in-out flow was developed. The spatiotemporal distribution of the SARS in-out flow was comprehensively explored. The characteristic parameter analysis of nodes was implemented on spatial and temporal scales, and the dynamic structural characteristics of the SARS transmission networks were also analyzed. As a novel research method for epidemic spread, studies based on the in-out flow and transmission network have better explained the spread patterns caused by the location change and character transformation of individuals and the interaction mechanism between regions during the spread process, and this research better detected and discovered potential spatial-temporal evolution rules and characteristics of the epidemic spread.

## 1 Data

The SARS epidemiological investigation data in mainland China from 2002 to 2003 originally included individual attributes of confirmed infected cases, which contained gender, age, occupation, household registration, working unit/home address, onset location, reporting unit, onset time, treated time, and confirmed time of recovery, among other parameters. The original data were stored as data sheets, and the individual onset time, which was selected as temporal information, was directly applied for quantitative analysis in a Time-Date format. Three typical locations

(working unit/home address, onset location and reporting unit) were selected for modeling the SARS epidemic in-out flow; this information was stored in Text format in the original data, and the description scales were not uniform. Therefore, the spatial data processing first required that the scales of the three locations should be converted into a consistent province or municipality, and second, the selected data had at least two integrated locations; thus, individuals whose data had two or three missing locations were eliminated. There were about 99.75% of original individual cases in mainland China selected in the final dataset, and the three location data were matched artificially and manually with geographical administrative maps with a scale of 1∶1000000.

Furthermore, 51.63% and 1.26% of the final selected cases had missing data for working unit/home address and reporting unit, respectively, and needed to be recovered, while all selected cases had complete onset location data. Few cases had missing information on other attributes, such as gender, age, and occupation. To maintain data integrity for the analyses, the recovery process was consistent in the regional attribute distributions and measured the relative influence of population distributions within the regions.

For some attributes with missing data, $n_i$ and $p_i$ were assumed to be the levels of existing effective data and population for a region $i$, respectively, and their corresponding relative ratios were calculated as $\rho_i=n_i/N$ and $\rho_{i,pop}=p_i/P$, where $N$ and $P$ were the total amounts of existing effective data and population for all regions, respectively. The parameter $\alpha$ was used to describe the relative adjustment between the existing effective data and population, and the ratio of data recovery for region $i$ and its accumulative ratio were calculated as

$$\theta_i = (1-\alpha) * \rho_i + \alpha * \rho_{i,pop}, \quad (1)$$

$$\Theta_i = \sum_{1}^{i} \theta_i . \quad (2)$$

A random number *rand*, which ranged from 0 to 1, was set for particular attributes with missing data; *rand* was determined to be within the interval of $[\Theta_{i-1}, \Theta_i]$, and certain information corresponding with $\Theta_i$ was used to recover missing attribute data. Considering the characteristic consistency of the spatial distribution of the original data, the adjustment parameter $\alpha$ was set as 0.2. Based on the above recovery process, the similarity ratio for the attribute of reporting unit was 99.99% because of the extremely small proportion of missing data, and the similarity ratio for the attribute of working unit/home address was 98.22% due to the relatively large proportion of missing data.

## 2 Methods

### 2.1 SARS in-out flow

During the epidemic transmission period, susceptible individuals changed their location information continuously after becoming infected. In several studies of epidemic transmission and spatial-temporal simulation, only a single variable of spatial information, such as onset location, was considered. Susceptible individuals had periods of being infected, exposed and treated, which corresponded with multiple spatial data, such as the infected, onset and treatment locations. In contrast with other epidemic models, the in-out flow model primarily focuses on the location transformation process of infected individuals during the epidemic transmission period, and this model explores the spread mechanism of epidemic transmission inputs and outputs between regions on various scales. Epidemic in-out flow can be defined by considering the three typical location data of individuals during an epidemic period: working unit or home address (*S1*), onset location (*S2*) and reporting unit (*S3*). *S1* is considered the main residence of individuals because the working unit and home address are the two most important locations. *S2* is used as the approximate infection location of the individuals. The exposure period of most infectious diseases is short, and the spatial-temporal information of infected individuals is difficult to collect; therefore, the onset location is used instead of the infection location. *S3* commonly represents the treatment location of the infected individuals and is collected using the last medical units.

Among most individuals, the main residence transforms to the infection location after the individual becomes infected, and the residence transforms into the onset location when symptoms appear. Finally, this location transforms to the reporting unit where the individuals receive treatment. During the epidemic period, individuals transform their location information from region to region, and for spatial regions, viral inputs and outputs between regions influence the spread process. In the logical definition of an epidemic in-out flow, the inputs of some regions, such as Beijing, in the province/municipality scale, represent the cases that have the treatment location that corresponds with Beijing but the residence is not Beijing, and the outputs are cases in which the residence is Beijing but the treatment location is not. Additionally, those who have the residence of Beijing and also receive treatment in Beijing are self-spread cases of Beijing. Moreover, considering the infection location of the individuals, the inputs and outputs could be categorized as primary and secondary types. The primary inputs and outputs are cases in which the infection location is Beijing and is not Beijing, respectively, and the secondary inputs and outputs are those in which the infection location is not Beijing and is Beijing, respectively. The epidemic in-out flow of Beijing is described by the following logical expressions:

$$\text{Input: S1} \neq \text{'Beijing' AND S3} = \text{'Beijing'} \begin{cases} \text{Primary: S2} = \text{'Beijing'}, \\ \text{Secondary: S2} \neq \text{'Beijing'}, \end{cases} \quad (3)$$

$$\text{Output: S1} = \text{'Beijing' AND S3} \neq \text{'Beijing'} \begin{cases} \text{Primary: S2} \neq \text{'Beijing'}, \\ \text{Secondary: S2} = \text{'Beijing'}. \end{cases} \tag{4}$$

The inputs and outputs of some regions indicated that the information and material flows of the epidemic spread could end and start in a particular region. The in-out flows of other provinces and municipalities could be similarly defined as Beijing. The above epidemic in-out flows focus on the spatial information that included the residence, onset and treatment locations and reflect the characteristics and mechanism of the epidemic spread on the spatial scale. During the epidemic period, different regions had various inputs and outputs during the time sequence, and the input and output flows dynamically indicated the spread mechanism between regions caused by the location changes of individuals corresponding with character transformation.

## 2.2 Epidemic transmission network

Compared with regular network and random network models [16,17], complex network models are more scientific and effective for modeling complex systems in the real world, and these models are more accurate in describing the various network characteristics of the real world. In particular, after models of small-world networks [18,19] and scale-free networks [20] were proposed, research on complex networks reached a climax. Based on the definition of an epidemic in-out flow, there were two types of input and output flows in mainland China during the SARS epidemic spread period. Different regions had various inputs and outputs in a time sequence, and the in-out flows of different regions comprised directed and weighted transmission networks that were dynamically changing. Two types of SARS transmission networks, including an input network and output network, described the transformation directions and weights of infected cases and indicated the dynamic interactions between provinces or municipalities during the epidemic spread process.

According to the epidemiological survey data, the input and output cases of the regions could be determined by the time sequence and on different scales. Each input or output flow has two location parameters: the start location and the end location. The two types of transmission networks of epidemic in-out flow could be defined according to the input and output flow results. $N_i$ is used as a certain node in the transmission network that corresponds to the spatial regions. $E_j$ is defined as a certain directed edge connecting the two nodes that describe the input or output flows between two regions. The two start and end nodes connected by $E_j$ are $N_s(j)$ and $N_e(j)$. In the input flow network, the direction of edge $E_j$ is defined from $N_s(j)$ to $N_e(j)$, and the weight of edge $E_j$ is the input cases from $N_s(j)$ to $N_e(j)$. Similarly, in the output flow network, the direction of edge $E_j$ is from $N_e(j)$ to $N_s(j)$, and its weight is the output cases from $N_e(j)$ to $N_s(j)$. For all of the provinces in mainland China, the input and output cases from each node to another node in a time sequence could be obtained based on the definition of the in-out flow and epidemiological data, and moreover, the corresponding input and output networks could also be dynamically built.

## 2.3 Network characteristic parameters

To understand the two types epidemic in-out flow transmission networks, some typical characteristic parameters of the complex network were applied for network analysis, including degree, shortest path, distance and betweenness centrality of nodes, average degree, degree distribution, average distance and diameter of network, as well as the clustering coefficient of the nodes and networks.

For a certain node $N_i$, its degree indicates the count of other nodes that have connections with $N_i$ or the count of edges that have $N_i$ as one of their two nodes; and in directed networks, there are two types of degrees, the in-degree and the out-degree, for which the mean $N_i$ is the end node or the start node. $\langle k \rangle$ is defined as the average degree of network, which represents the average degree values of all nodes. $p(k)$ is the degree distribution of the network, which represents the count ratio of the nodes that have a degree value $k$ to the total nodes; furthermore, $p(k)$ describes the probability that the degree value of a random node in the network equals $k$. According to different degree distributions, the networks could be classified into different types [21]. For example, the degree distribution of random networks follows a Poisson distribution, and scale-free networks follow a power-law degree distribution. Furthermore, a homogeneous network and inhomogeneous network could also be defined according to degree distributions.

The shortest path between two nodes in a network represents one of the paths connecting the two nodes with the least edge count. $d_{ij}$ is defined as the distance of nodes $i$ and $j$, which represents the edge count of the shortest path between nodes $i$ and $j$. The diameter of the network represents the maximum of distances between any two nodes in a network, and is described as

$$D = \max_{i,j} d_{ij}. \tag{5}$$

$L$ is the average distance of the network, which represents the average value of distances between any two nodes in network, and is defined as

$$L = \frac{1}{N(N+1)/2} \sum_{i \geq j} d_{ij}, \tag{6}$$

where $N$ is the whole node count in the network.

$C_i$ is defined as the clustering coefficient of node $i$, which is the ratio of the practical edge count to the utmost probable edge count between the nodes connected to node $i$. Assuming there are $k_i$ nodes connected to node $i$, and that the practical edge count between those $k_i$ nodes is $A_i$, the clustering coefficient of node $i$ can be described as [18]:

$$C_i = \frac{A_i}{k_i(k_i - 1)/2}. \tag{7}$$

Correspondingly, the clustering coefficient of the network describes the connection situation between the nodes connected to the same node and reflects the trend of aggregation clustering of the nodes in a network. The average value of clustering coefficients of all nodes [18] is implemented as

$$C = \frac{1}{n}\sum_i C_i. \tag{8}$$

The betweenness centrality of the nodes is another important parameter that describes the clustering characteristic of the nodes. $BC_i$ is defined as the betweenness centrality of node $i$, which represents the count of the shortest paths with node $i$ as an intermediate node, and can be calculated as

$$BC_i = \sum_{m \neq i \neq n} \frac{\sigma_{mn}(i)}{\sigma_{mn}}, \tag{9}$$

where $\sigma_{mn}$ is the count of all the shortest paths from node $m$ to node $n$; and $\sigma_{mn}(i)$ is the count of all the shortest paths from node $m$ to node $n$ that have node $i$ as an intermediate node.

Furthermore, in our transmission networks of epidemic in-out flow, the edges connecting the nodes have both directions and weights. The in-degree and out-degree distribution could help describe the direction of the epidemic spread between regions, but the standard expression of the clustering coefficient can only describe the information flow of the epidemic spread between the nodes. To reflect the material flow of the epidemic spread, an improved expression of clustering coefficient is advanced as follows:

$$C_i^* = \sum_j^{A_i} Cas_j \Big/ \sum_j^{A_N} Cas_j, \tag{10}$$

where $A_N$ is the count of all edges in the network and $Cas_j$ is the material flow of the edge $j$, which represents the count of input or output cases in the transmission network of epidemic in-out flow. The improved clustering coefficient $C_i^*$ describes the clustering situation of the material flow between the nodes connected to node $i$. The corresponding network clustering coefficient $C^*$ directly reflects the small-world characteristic of the epidemic in-out flow.

## 3 Results and interpretation

### 3.1 Spatiotemporal distribution of SARS in-out flow

The levels of input, output and self-spread cases in the provinces of mainland China were calculated based on the definition of the in-out flow and the SARS epidemiological investigation data. The total number of self-spread cases was 2625, and the number of input/output cases was 2825, among which primary input/output cases were the majority, while secondary cases accounted for only a small proportion, indicating that most of the input and output cases received treatment at the same location as the onset location, and therefore, the viral spread range caused by the location transformation of the individuals was maintained to a small extent. The spatial distribution of the cumulative input cases of the provinces is shown in Figure 1(a), which indicates that the distribution of input cases had significant clustering characteristics consistent with that of self-spread cases, with Beijing and Guangdong as clustering centers. The difference between Beijing and Guangdong was that the provinces around Beijing had a certain number of input and self-spread cases, while the provinces around Guangdong were less affected. Figure 1(b) illustrates the spatial distribution of the cumulative output cases in the provinces, indicating that the output cases covered most of the eastern provinces of mainland China and some of the western provinces, such as Sichuan. A majority of the output cases were from Beijing and Guangdong, but a number of output cases were in the provinces surrounding Beijing and Guangdong.

Five provinces with significantly large numbers of input/output cases were selected—Shanxi, Guangdong, Inner Mongolia, Hebei and Beijing—and the temporal changes of their cumulative input, output and self-spread cases were calculated, as shown in Figure 2. Some findings could be obtained: (1) Beijing was one of the most hard-hit provinces in the SARS epidemic, but its input cases did not significantly increase until the mid-term epidemic period, and its overall number accounted for a lower value in final; concerning the provinces surrounding Beijing, such as Hebei, Shanxi and Inner Mongolia, the changes in the number of input cases were consistent, and their cumulative numbers accounted for a larger value compared with that of Beijing; (2) the cumulative input cases in Guangdong increased late and rapidly reached a higher value until the later epidemic period; (3) the output cases of the five provinces started to increase significantly in mid-January and maintained a slow growth at the beginning of the SARS epidemic, but there was a significant trend of rapid growth in the mid-late period; (4) compared with Beijing, the cumulative number of output cases in Guangdong grew slowly at the beginning, but in the late period, the number of output cases increased so rapidly that the cumulative number was similar to that of Beijing at the end of the period; (5) Guangdong was a good example of a province with significant growth of self-spread cases, with sporadic self-spread cases in early January, and rapid growth occurred during the early epidemic and continued for the entire period; and (6) the self-spread cases were a notable feature of the SARS epidemic in Beijing;

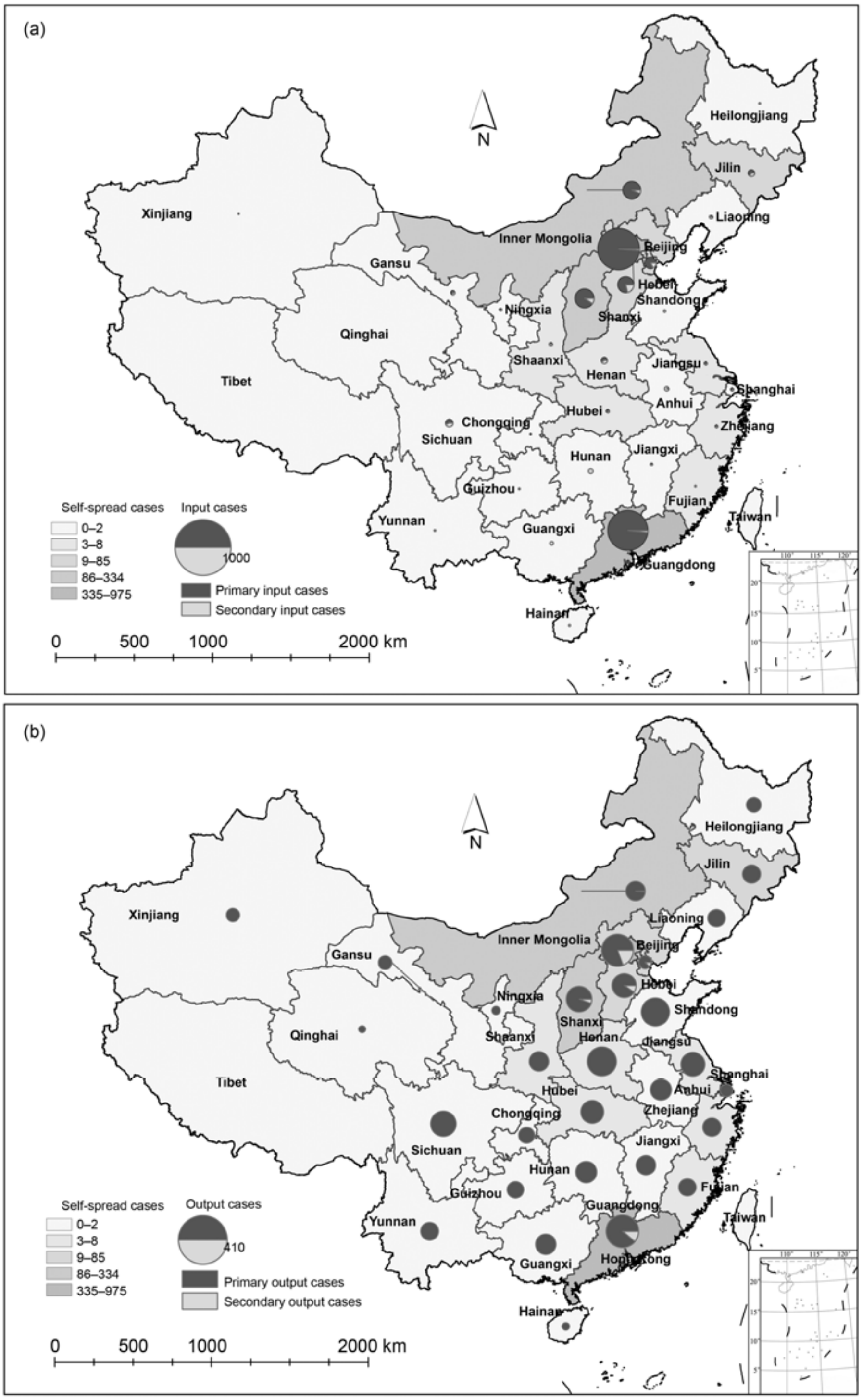

**Figure 1** Spatial distribution of SARS in-out flow: (a) input cases; (b) output cases. Xinjiang: Xinjiang Uygur Autonomous Region; Tibet: Tibet Autonomous Region; Guangxi: Guangxi Zhuang Autonomous Region; Inner Mongolia: Inner Mongolia Autonomous Region; the same below.

however, these cases appeared in the late epidemic, and the number of cases increased to a high value, with rapid growth.

## 3.2 Degree analysis of nodes and network

The input and output flow networks with the provinces/municipalities as nodes were built based on the in-out flows of the SARS epidemic from November 30, 2002 to May 10, 2003 in mainland China. The input flow network showed the input cases from other nodes to a particular node, and the output flow network indicated the output cases from a particular node to the other nodes, while the weight

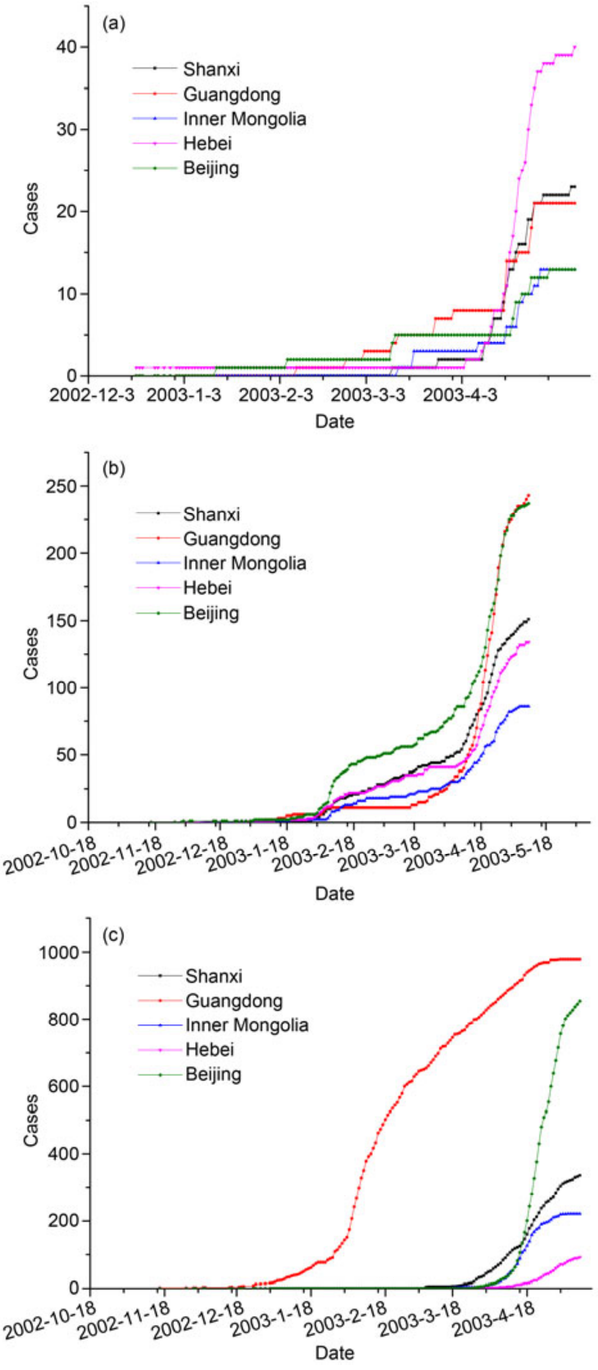

**Figure 2** Temporal changes of SARS in-out flow in the provinces of Shanxi, Guangdong, Inner Mongolia, Hebei and Beijing: (a) input cases; (b) output cases; (c) self-spread cases.

values of the edges indicated the statistical number of input/output cases. In the input and output flow networks, the values of the in-degree and out-degree of the nodes were calculated, indicating the levels of the input/output risk of the nodes. As illustrated in Figure 3, the spatial distribution of the node in-degree in the input flow network showed that the overall connection density was not high because there was only a small number of edges (84), and most of the weight values of the connecting edges were as low as 10 or less, except for the weight values of the edges between the central provinces (Beijing and Guangdong) of the SARS epidemic. Additionally, a few provinces surrounding these central provinces were at a level of 10–33. Furthermore, the distributions of the in-degree values of the nodes did not show significant spatial clustering characteristics. In detail, the in-degree values of Beijing and Guangdong were highest at 21 and 22, respectively, and the higher values were those of a few surrounding provinces, with values of 10–20, while the in-degree values of the other provinces were less than 10. The spatial distribution of the node out-degree in the output flow network is shown in Figure 4. The corresponding overall connection density was very high because there were as many as 265 edges in the output network, and the weight values of a certain number of edges were above 50, while the weight values of the edges from Beijing to Guangdong and Guangdong to Beijing were very high at 90 and 121, respectively. The distribution of the node out-degree had significant characteristics of spatial clustering, with Beijing and Guangdong as the clustering centers, and Sichuan in the west also had an impressive out-degree value. Some findings obtained based on the above analysis were as follows: (1) the SARS transmission network of mainland China had significant characteristics of spatial clustering in the output flow and two clustering centers: Beijing and Guangdong; (2) there were a small number of edges and low weight values, and there was no distinct clustering characteristics in the input flow network, which indicated that there was only a small range of risk spread caused by infected cases from onset to treatment, and control measures for infected cases, such as isolation, were remarkably effective; and (3) Sichuan had a particularly large range of output spreading, which indicated that it was necessary to implement priority control measures in provinces with mainly an output floating population during the SARS epidemic.

Five provinces (Shanxi, Guangdong, Inner Mongolia, Hebei and Beijing) with a significantly large number of both in-degree and out-degree were selected in the SARS transmission networks of input flow, and the output flow, temporal changes of node degree, in-degree and out-degree in those provinces were analyzed, as illustrated in Figure 5. The results suggested that (1) the out-degree of Guangdong in the input flow network appeared as early as the beginning of January 2003, and its corresponding in-degree began in early February, indicating that Guangdong was the early original source of the SARS epidemic, from which the infections in many other provinces were at an early time ; (2) the in-degree and out-degree of Beijing in the input flow network appeared to occur as late as in early-mid March, and the in-degree and out-degree of Beijing's three surrounding provinces (Shanxi, Hebei and Inner Mongolia) also appeared late, indicating that control measures had taken effect in the early-mid period of the SARS epidemic in regions centered in Beijing, while the input cases from

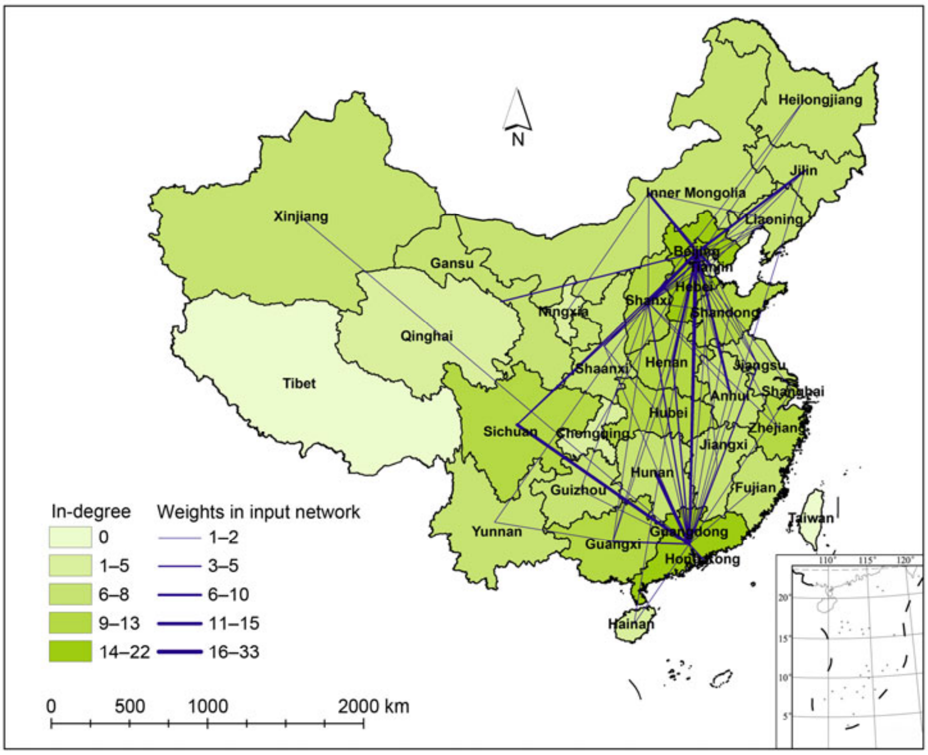

**Figure 3** Spatial distribution of node in-degree in input flow network.

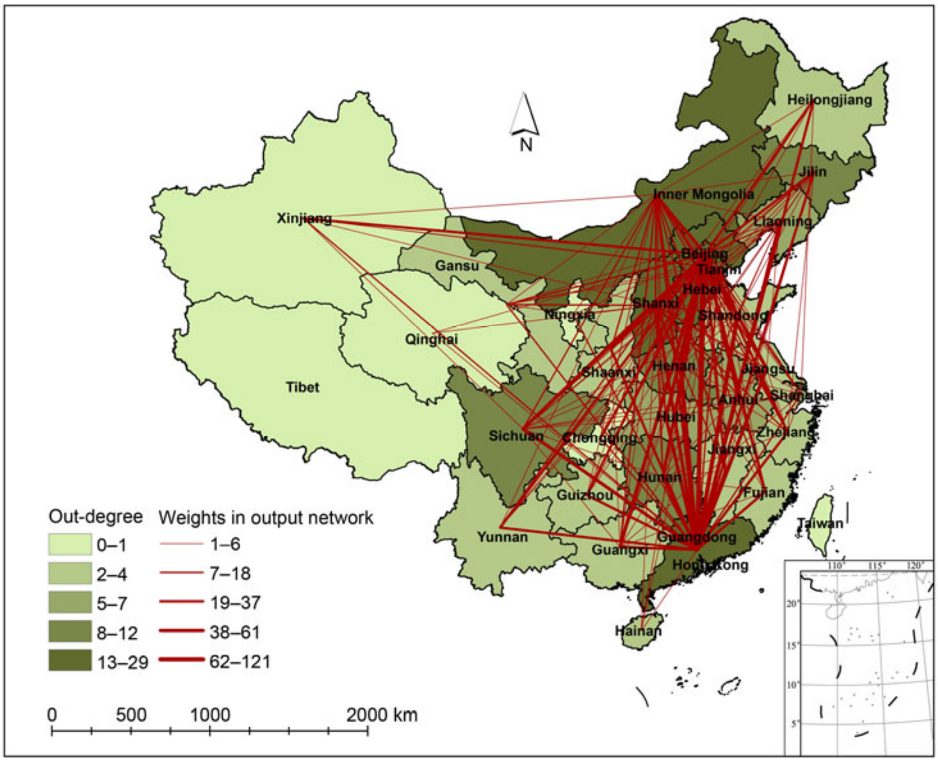

**Figure 4** Spatial distribution of node out-degree in output flow network.

other provinces increased dramatically in the mid-late epidemic period due to the substantial increase of the range of epidemic spread; (3) the in-degree and out-degree of Guangdong in the output flow network appeared early, and the in-degree increased to a maximum value in early February and was then maintained at the same value, indicating that

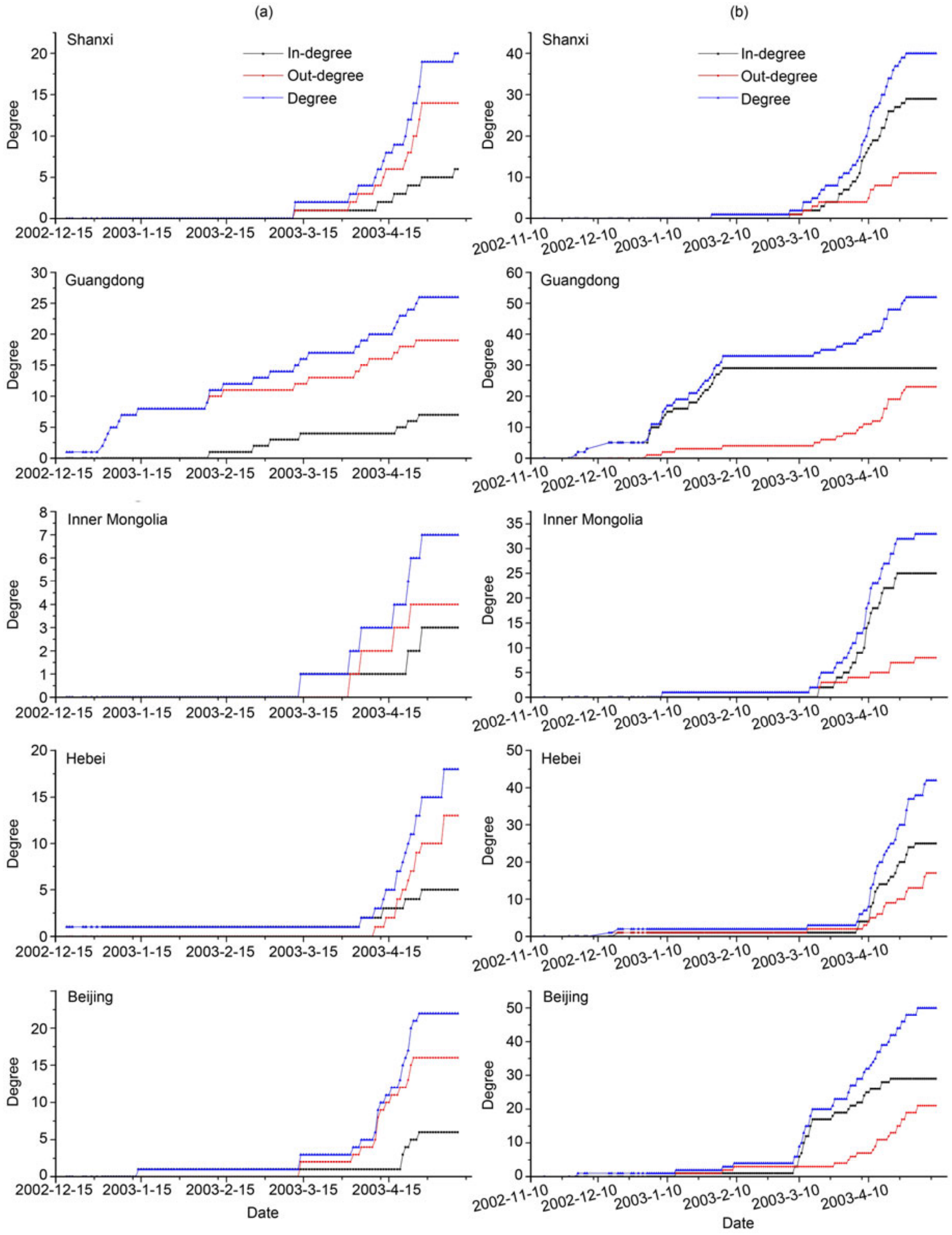

**Figure 5** Temporal changes of node degree/in-degree/out-degree Shanxi, Guangdong, Inner Mongolia, Hebei and Beijing. (a) Input flow network; (b) output flow network.

Guangdong was the primary origin of the SARS epidemic in the early period and that there were continuous output cases from Guangdong to the other provinces throughout the entire epidemic period; and (4) in the output flow network, the in-degree and out-degree of Beijing and its three surrounding provinces (Shanxi, Hebei and Inner Mongolia) appeared late and gradually spread to the national level during the mid-late period. The following interpretation was obtained from the above comprehensive analysis: Guangdong and Beijing were the two most severely affected provinces during the SARS epidemic in mainland China, but the characteristics and features of their epidemic spread were essentially different from each other. In particular, Guangdong was the original source of the SARS epidemic during the early-mid period and continued to output infections to other provinces throughout the whole epidemic period, but no clustering area formed with Guangdong as the center. However, although there was no obvious interaction of the input and output flows between Beijing and the other countrywide provinces until the mid-late period, Beijing had a distinct high-level capacity for spatial spreading and formed a clustering area for the SARS epidemic with its surrounding provinces (Shanxi, Hebei and Inner Mongolia).

### 3.3 Characteristic parameter analysis of nodes

The clustering coefficient and betweenness centrality are two important indicators that reflect the clustering characteristics of a network structure, and based on the constructed transmission network of the SARS in-out flow, the cumulative values of the clustering coefficient and betweenness centrality of nodes were calculated by code programming. In the input flow network, with its low connection density, the values of the betweenness centrality of most nodes were less than 100, and the values of the clustering coefficient of many nodes were 0, indicating that there were no significant clustering characteristics between nodes in the input flow network. However, there were a certain number of cold nodes with clustering coefficient values equal to 1, mainly because the number of nodes connected to nodes with a full connection load was too small to reflect the clustering characteristics. In the output flow network, with its distinctly high connection density, the statistical results of the node clustering coefficient could directly characterize the clustering feature; in addition, the betweenness centrality of most nodes was high. The combination analysis of the spatial distributions of the clustering coefficient and betweenness centrality in the input flow network and output flow network, which are shown in Figures 6 and 7, respectively, indicated that (1) the distribution of the clustering coefficient in the input flow network had no significant clustering characteristics and the clustering coefficient of hotspot nodes of the SARS epidemic, including Beijing and Guangdong, was also not high, indicating that although there were several other nodes from which the input cases of hotspots nodes arose, these source nodes were too cold to form a cluster with each other; (2) in the output flow network, the distribution of the clustering coefficient of the nodes had significant clustering characteristics, and the connection density between the nodes connected to Beijing and Guangdong was very high, while the distribution of the nodes connected to Beijing and Guangdong was relatively concentrated mainly in the north, east and southwest of mainland China; (3) in the input flow network, the betweenness centrality distribution was also concentrated mainly in Guangdong and Beijing with its surrounding provinces showing that the spatial floating of the input cases had some clustering characteristics; and (4) several provinces surrounding Beijing and Guangdong formed a distance clustering trend in the output flow network, indicating that the spatial floating of output cases mainly covered a range between Beijing, Guangdong and their surrounding provinces.

Shanxi, Guangdong, Inner Mongolia, Hebei and Beijing were selected for an analysis of temporal clustering coefficient and betweenness centrality changes of the nodes during the SARS epidemic period. As illustrated in Figure 8, the results suggested that (1) there were phases with unreasonably high-value clustering coefficients in both the input flow and output flow networks, and this was mainly due to no extensive interactions occurring between the cold nodes during the early-mid period of the SARS epidemic; (2) in the input flow network, the clustering coefficients of the two hotspot centers (Beijing and Guangdong) were not significant until mid-late March, indicating that there were no interactions between the nodes from which the infected cases of Beijing and Guangdong came during the early-mid period of the SARS epidemic; prevention/control measures should also be focused on these provinces with connections to hotspot nodes due to the appearance of input and output interactions between these nodes during the mid-late period; (3) in the output flow network, Guangdong appeared to have a clustering coefficient in early January, showing that the other nodes with output cases from Guangdong had interactions since the early epidemic period; however, Beijing did not appear to have a clustering coefficient until early March, with a high initial value, because there had been extensive interaction between most nodes since the mid-epidemic period; (4) there was a betweenness centrality value in Guangdong since early January in the input flow network, which was further evidence that Guangdong was the original source of the SARS epidemic during the early period. The betweenness centrality value of Guangdong continued to increase, indicating that Guangdong carried the most information of input flow throughout the entire period; in contrast, Beijing and its surrounding Hebei, Shanxi and Inner Mongolia carried only a small part of the information of input flow during the early period and gradually increased through the late period; (5) in the output flow network, the betweenness centrality value in Guangdong appeared early, with a high value, and rapidly increased to the maximum value without decreasing until late in the epidemic, indicating that Guangdong began to output cases to many provinces since the early epidemic period, and that its output flow rapidly covered an almost nationwide scope; in the mid-late period, after another hotspot clustering area was formed in Beijing and its surrounding provinces, where the influence sphere of the output flow from Guangdong gradually weakened; and (6) the betweenness centrality value

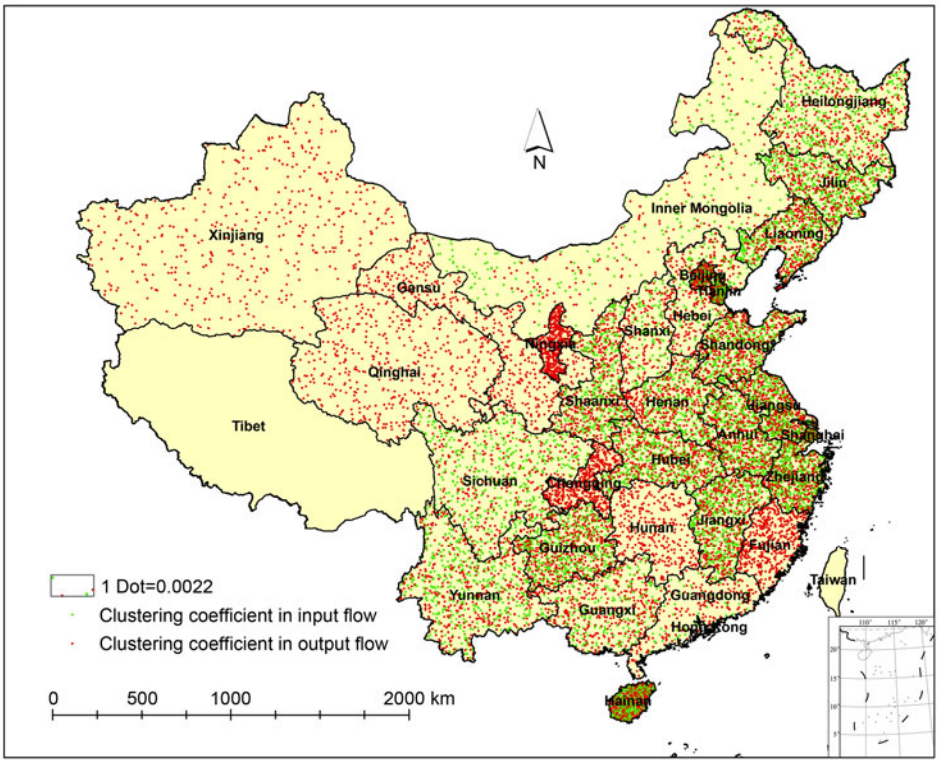

**Figure 6** Spatial distribution of clustering coefficient of nodes.

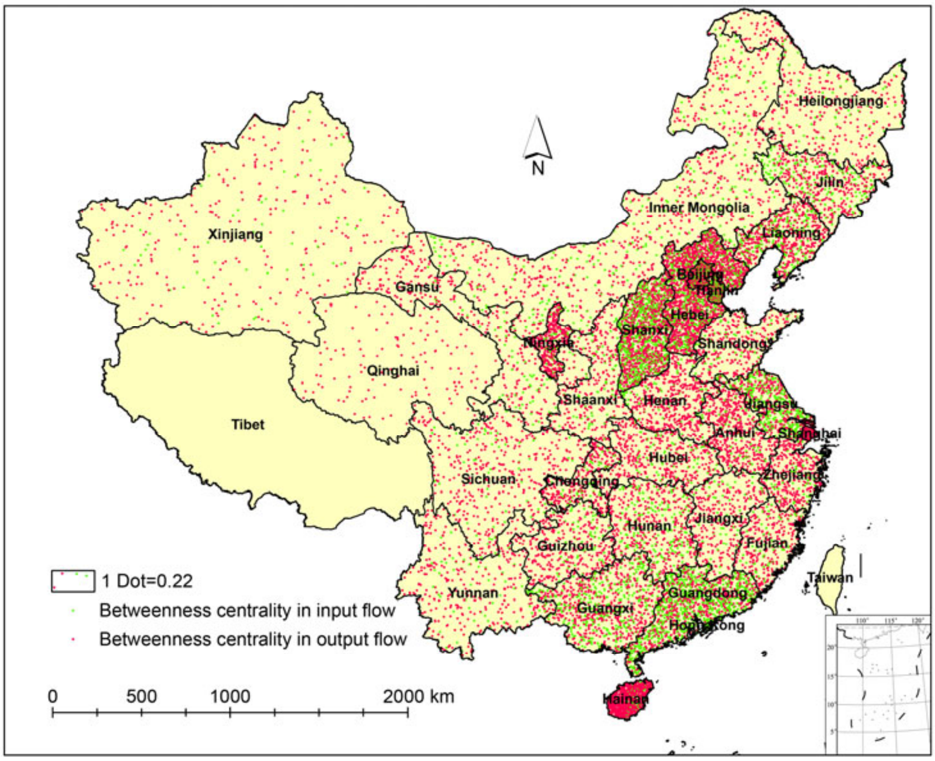

**Figure 7** Spatial distribution of betweenness centrality of nodes.

of Beijing appeared during the early period and increased gradually in the output flow network while forming a larger sphere of influence, and the nodes surrounding Beijing maintained the same output flow spread level, while during the late period, Hebei had an even higher level of spread range than Beijing, indicating that control measures for the

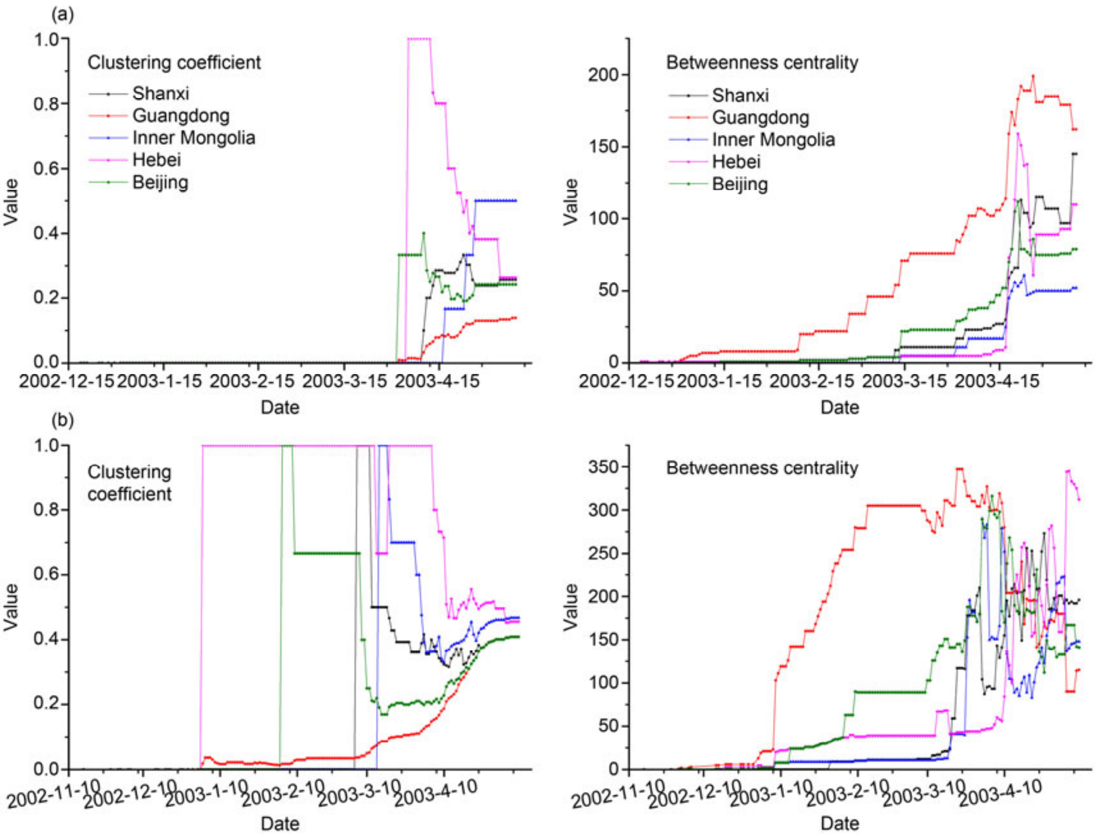

**Figure 8** Temporal changes of clustering coefficient and betweenness centrality in the provinces of Shanxi, Guangdong, Inner Mongolia, Hebei and Beijing. (a) Input flow network; (b) output flow network.

nodes surrounding Beijing should be strengthened during the later epidemic, especially for the Hebei Province.

## 3.4 Structural characteristics of input flow and output flow networks

During the SARS epidemic, the input flow and output flow networks formed by the input/output interactions between provinces were dynamic, directed and weighted, and the network structural characteristics gradually changed during the time sequence. The temporal changes of the structural characteristics of the two types of networks were analyzed in a one-day time interval. Figure 9 illustrates the temporal changes of the diameter, average distance and average degree of the input/output flow networks. The results showed that (1) the overall connection density of the input flow network was low, and its diameter, average distance and average degree were at a low value, with a slow integrated increasing trend; (2) in the input flow network, the maximum values of the average degree and average distance were 2.54 and 4.94, respectively, indicating that the number of nodes with input flow spreading was small and that the spreading range was also low; (3) the maximum value of the diameter in the input flow network was 20, indicating that the spreading range of the input flow belonged to a middle level on the national scale; (4) in the output flow network with high connection density, the average degree value appeared early and expanded rapidly to a maximum value of 14 during the mid-late period, indicating that the number of objects affected by the output flow was large; (5) the average distance value in the output flow network appeared early in the epidemic, increased rapidly to a maximum of 14 and then had a small irregular decreasing trend, indicating that the spreading intensity of the output flow was very strong during the early-mid period and effectively controlled to a certain extent, when control measures were strengthened later in the epidemic; and (6) in the output flow network, the diameter value rapidly increased to a maximum value of 41 and showed a clear decreasing trend in the late epidemic period, indicating that the spreading range of the output flow covered the nation in only the mid-epidemic period and was apparently controlled until the mid-late period with strengthened control measures.

Further analysis on the temporal clustering coefficient and betweenness centrality changes in the input/output flow networks were implemented and shown in Figure 10. The analyses indicated that (1) in the input flow network, with its weak clustering characteristics, the clustering coefficient appeared at the end of March, which was at the same time as when the betweenness centrality value significantly increased, indicating that the spreading range of the input flow showed overall stochastic features without significant

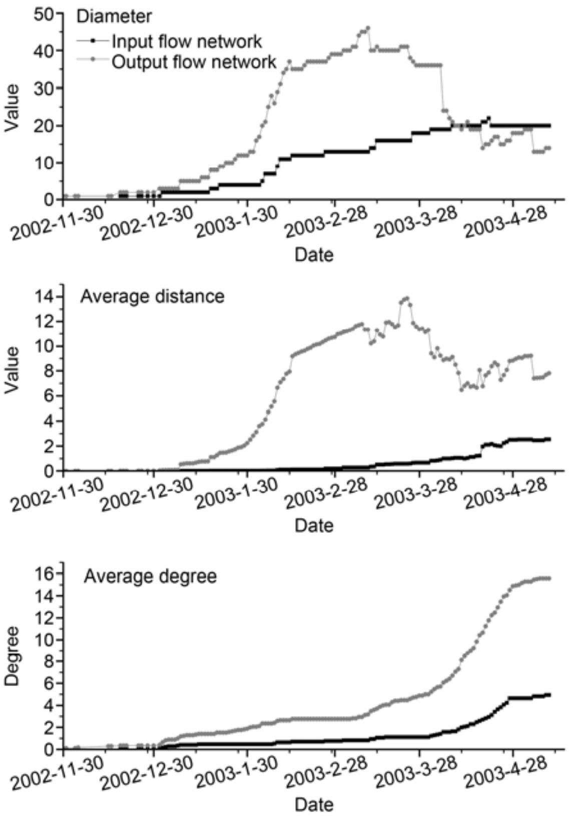

**Figure 9** Temporal changes of diameter, average distance and average degree of input/output flow networks.

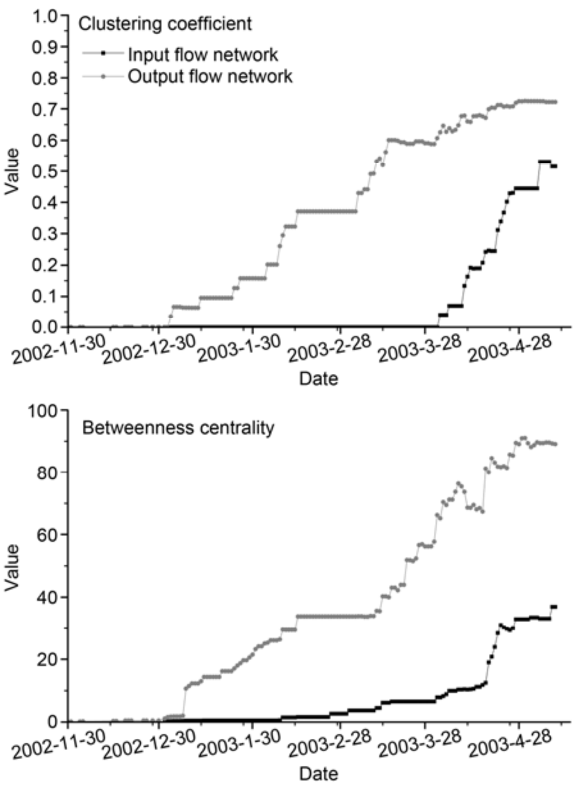

**Figure 10** Temporal changes of clustering coefficient and betweenness centrality of input/output flow networks.

clustering characteristics, even late in the epidemic, and (2) the clustering coefficient value in the output flow network appeared at an early stage, continued growing and reached a maximum value of 0.72; similarly, the betweenness centrality value had the same developing trend and reached a maximum value of 89.62, indicating that there was significant clustering characteristics in the output flow network, and the characteristics gradually became increasingly intense during the entire SARS epidemic period.

## 4 Conclusion and discussion

The spread of infectious diseases is due to interactions in a human-earth environment and forms a complex system during an epidemic period. The exploration and study of the spatiotemporal characteristics and patterns of epidemic spread extensively aid the understanding of the spread mechanism and provide theoretical support for the prevention and control of future novel epidemics. Extensive research on epidemic spread mechanisms has been conducted in pathology, epidemiology, medical statistics, spatial information science, sociology, and anthropology, among other fields, and the results have provided detailed explanations regarding spread mechanisms, spatiotemporal distribution and prevention/control measures. However, the systematic understanding and explanation of spatiotemporal change patterns of the individual susceptible-infected-symptomatic-treated-recovered epidemic process and dynamics that direct the transmission network between individuals and regions caused by interactions of information and material flow are still lacking. In this paper, the SARS epidemic in mainland China in 2002–2003 was used as an example. Based on key data regarding individual location change in the process of being infected, disease onset, receiving treatment and recovery, the concept of in-out flow was defined to effectively explain the spatiotemporal evolution pattern of the individual location transformation in the SARS epidemic and characteristics of information and material flow in the dynamic transmission network caused by the input/output interactions between regions. The transmission network of the SARS epidemic was built based on the in-out flow, and the spatiotemporal distribution of the SARS in-out flow, the spatial distribution and temporal change of node characteristic indicators, and the network structure characteristics were comprehensively and systematically implemented, resulting in a series of new conclusions. Future research based on these two new perspectives, including the epidemic in-out flow and transmission network, may help explain the spatiotemporal spread pattern of infectious diseases and provide new study ideas and practical implications, including results revealing the characteristics and laws of the SARS epidemic in mainland China.

Discussion and proposed future work are as follows: (1) the proposed definition of the SARS epidemic in-out flow was primarily based on three individual locations (working unit/home address, onset location and reporting unit), and to provide a more accurate description of the individual susceptible-infected-symptomatic-treated-recovered epidemic process, the location data of the in-out flow could be further redefined to be more exact; (2) the SARS transmission network model can be optimized, for instance, by adding a ring structure to the network to consider the self-spread node cases, and the two input flow and output flow networks could be merged as an integrated network considering the bidirectional and multi-weighted edge characteristics; (3) the weights of the nodes could be diversified by considering population density, traffic level, and socio-economic indicators of the nodes; and (4) the study scale could be further redefined by building a transmission network model with cities or counties as nodes, which more accurately analyze the network structure and explore additional potential characteristics of the SARS epidemic.

*This work was supported by the National Natural Science Foundation of China (40871181 and 41101369), the Key Knowledge Innovative Project of Chinese Academy of Sciences (KZCX2-EW-318), the Jiangxi Province Natural Science Foundation (20114BAB215024), the Natural Science Youth Foundation of Jiangxi Province Office of Education (GJJ11073), the Open Foundation of Key Laboratory of Poyang Lake Wetland and Watershed Research, Ministry of Education (PK2010001), and the Jiashan Science and Technology Project "Intelligent Management System and Application of Urban Humans-Things Based on Virtual Geographic Environment" (2011B04).*

1 Jia N, Tsui L. Epidemic modelling using SARS as a case study. North Amer Act J, 2005, 9: 28–42
2 Dye C, Gay N. Epidemiology modeling the SARS epidemic. Science, 2003, 300: 1884–1885
3 Shi Y L. Stochastic dynamic model of SARS spreading. Chin Sci Bull, 2003, 48: 1287–1292
4 Anderson R M, Fraser C, Ghani A C, et al. Epidemiology, transmission dynamics and control of SARS: The 2002–2003 epidemic. Philos Trans R Soc Lond B Biol Sci, 2004, 359: 1091–1105
5 Lipsitch M, Cohen T, Cooper B, et al. Transmission dynamics and control of severe acute respiratory syndrome. Science, 2003, 300: 1966–1970
6 Pang X, Zhu Z, Xu F, et al. Evaluation of control measures implemented in the severe acute respiratory syndrome outbreak in Beijing, 2003. JAMA, 2003, 290: 3215–3221
7 Cao Z D, Zeng D J, Zheng X L, et al. Spatiotemporal evolution of Beijing 2003 SARS epidemic. Sci China Earth Sci, 2010, 53: 1017–1028
8 Fang L Q, de Vlas S J, Feng D, et al. Geographical spread of SARS in mainland china. Trop Med Int Health, 2009, 14(Suppl 1): 14–20
9 Wang J F, Christakos G, Han W G, et al. Data-driven exploration of "spatial pattern-time process-driving forces" associations of SARS epidemic in Beijing, China. J Public Health (Oxf), 2008, 30: 234–244
10 Wang J F, Mcmichael A J, Meng B, et al. Spatial dynamics of an epidemic of severe acute respiratory syndrome in an urban area. Bull World Health Organ, 2006, 84: 965–968
11 Meng B, Wang J F, Liu J, et al. Understanding the spatial diffusion process of severe acute respiratory syndrome in Beijing. Public Health, 2005, 119: 1080–1087
12 Gong J H, Sun Z L, Li X W, et al. Simulation and analysis of control of Severe Acute Respiratory Syndrome (in Chinese). J Remote Sens, 2003, 7: 260–265
13 Gong J H, Zhou J P, Xu S, et al. Dynamics model and multi-agent based simulation of SARS transmission (in Chinese). J Remote Sens, 2006, 10: 829–835
14 Lin G J, Jia X, Ouyang Q. Predict SARS infection with the small world network model (in Chinese). J Peking Univ (Health Sci), 2003, 35(Suppl): 66–69
15 Small M, Tse C K. Small world and scale free model of transmission of SARS. Int J Bifurcation Chaos, 2005, 15: 1745–1755
16 Erdős P, Rényi A. On the evolution of random graphs. Pub Math Inst Hungarian Acad Sci, 1960, 5: 17–61
17 Erdős P, Rényi A. On the strength of connectedness of a random graph. Acta Math Sci Hungary, 1961, 12: 261–267
18 Watts D J, Strogatz S H. Collective dynamics of /′small-world/′networks. Nature, 1998, 393: 440–442
19 Watts D J. Networks, dynamics, and the small world phenomenon model. Phys Lett A, 1999, 263: 341–346
20 Barabasi A L, Albert R. Emergence of scaling in random networks. Science, 1999, 286: 509–512
21 Amaral L A, Scala A, Barthelemy M, et al. Classes of small-world networks. Proc Natl Acad Sci USA, 2000, 97: 11149–11152

**Open Access** This article is distributed under the terms of the Creative Commons Attribution License which permits any use, distribution, and reproduction in any medium, provided the original author(s) and source are credited.
